# Benzimidazole inhibitors of the protein kinase CHK2: Clarification of the binding mode by flexible side chain docking and protein–ligand crystallography

**DOI:** 10.1016/j.bmc.2012.09.024

**Published:** 2012-11-15

**Authors:** Cornelis Matijssen, M. Cris Silva-Santisteban, Isaac M. Westwood, Samerene Siddique, Vanessa Choi, Peter Sheldrake, Rob L.M. van Montfort, Julian Blagg

**Affiliations:** a Cancer Research UK Cancer Therapeutics Unit, Division of Cancer Therapeutics, Institute of Cancer Research, Haddow Laboratories, Sutton, Surrey SM2 5NG, UK; b Division of Structural Biology, Institute of Cancer Research, Chester Beatty Laboratories, Chelsea, London SW3 6JB, UK

**Keywords:** ADP, adenosine diphosphate, ATM, ataxia telangiectasia mutated, ATP, adenosine triphosphate, CHK2, checkpoint kinase 2, GOLD, genetic optimisation for ligand docking, GST, glutathione S-transferase, KD, kinase domain, MOE, molecular operating environment, PARP, poly ADP-ribose polymerase, PDB, protein data bank, PLIF, protein ligand interaction fingerprints, SAR, structure activity relationship, SIFt, structural interaction fingerprints, Kinase, CHK2, Flexible docking, Crystallography, Inhibitor

## Abstract

Two closely related binding modes have previously been proposed for the ATP-competitive benzimidazole class of checkpoint kinase 2 (CHK2) inhibitors; however, neither binding mode is entirely consistent with the reported SAR. Unconstrained rigid docking of benzimidazole ligands into representative CHK2 protein crystal structures reveals an alternative binding mode involving a water-mediated interaction with the hinge region; docking which incorporates protein side chain flexibility for selected residues in the ATP binding site resulted in a refinement of the water-mediated hinge binding mode that is consistent with observed SAR. The flexible docking results are in good agreement with the crystal structures of four exemplar benzimidazole ligands bound to CHK2 which unambiguously confirmed the binding mode of these inhibitors, including the water-mediated interaction with the hinge region, and which is significantly different from binding modes previously postulated in the literature.

## Graphical abstract

Unconstrained rigid docking, flexible side chain docking and protein crystal structure determinations reveal a water-mediated hinge binding mode for a series of benzimidazole ligands of the protein kinase CHK2. This binding mode is different from those previously postulated in the literature and may provide a useful approach to selective small molecule inhibitor design.

## 1 Introduction

Checkpoint kinase 2 (CHK2) is a serine/threonine protein kinase and a component of the ATM-mediated response to double-strand DNA breaks. It has been postulated that selective inhibition of CHK2 could increase the efficacy of genotoxic cancer therapies in a p53 mutant background by modulating resistance pathways and may also be radioprotective to normal p53 wild-type tissues.^1,2^ Recent results demonstrate that selective inhibition of CHK2 in combination with PARP inhibition could also be therapeutically beneficial in cancer therapy.^3^ Small molecule inhibitors of CHK2 have been disclosed including the staurosporine analogue UCN-01,^4,5^ an indoloazepine derivative of hymenialdisine,^6^ isothiazole carboxamidines,^7,8^ bisguanylhydrazones,^9,10^ the dual CHK1/CHK2 inhibitor AZD7762^11^ as well as 3,5-diaryl-2-aminopyridines^12^ and 2-(quinazolin-2-yl)phenols, of which the potent and selective chemical tool CCT241553 is an example.^13,14^

**Figure 1:**
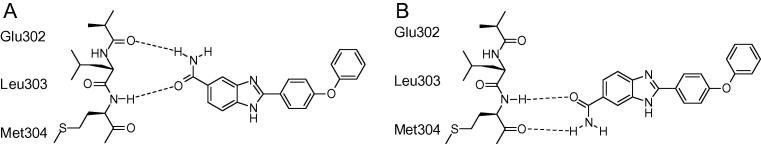
Two postulated binding modes of benzimidazole CHK2 inhibitors: (A) interaction with Glu302 and Met304 of the hinge; (B) interaction with Met304 of the hinge.

As part of our in-house drug discovery project, we were particularly intrigued by a published series of benzimidazole CHK2 inhibitors for which two closely related binding modes have been proposed, neither of which appeared entirely consistent with the reported SAR (vide infra).^15–17^ Benzimidazole is a common kinase inhibitor scaffold with compounds reported to inhibit NEK2,^18^ IKKε,^19^ GSK3β,^20^ and p38^21^ as well as CHK2.^15–17^ Analysis of the 52 protein kinase crystal structures in the protein databank (PDB)^22,23^ which incorporate a benzimidazole-containing ligand revealed multiple binding modes. In some of these the benzimidazole is acting as part, or all, of the hinge-binding motif^18–21^ whereas others indicate a scaffolding role without evidence for direct hinge binding.^24–26^ However, this set of protein–ligand structures does not contain any benzimidazole-based ligands bound to CHK2. The two closely related binding modes that have been proposed for the published class of benzimidazole CHK2 inhibitors both postulate that a pendant carboxamido substituent on the benzimidazole scaffold interacts directly with the hinge (Fig. 1).^15,16^ In one proposed binding mode, the carboxamide group forms two canonical hydrogen bonds with Glu302 and Met304 (Fig. 1A), whilst in the other, the carboxamide interacts exclusively with Met304 (Fig. 1B).^16^

Literature SAR includes analogues where the benzimidazole scaffold has been modified or replaced (Table 1) and has been proposed to be consistent with binding mode B.^15,16^ For example, the loss of affinity observed upon replacement of the 5-carboxamido group (compound **1**, Table 1) with a carboxylic acid, nitrile or nitro group, or upon methylation of the carboxamide (compounds **2**–**6**, Table 1) has been postulated to be consistent with binding of the 5-carboxamido group directly to the hinge. Preference for binding mode B was proposed based upon the potency of the primary sulphonamide (compound **7**, Table 1) in which interaction with the hinge through a bidentate H-bond donor–acceptor interaction to Met304 was proposed to place the polar sulphonamide in a more favourable hydrophilic environment.^15^ However, a substructure search of the sulphonamide motif in the PDB^22,23^ revealed no examples of ligands with a sulphonamide bound to the hinge region of a kinase. Moreover, significant loss of potency was observed upon methylation of either benzimidazole nitrogen (compounds **8** and **9,** Table 1) and upon replacement of the benzimidazole with the benzisoxazole or pyrazolopyrimidine scaffold (compounds **10, 11** and **12**, Table 1).^16^ In summary, existing biochemical SAR is consistent with an essential role for all four polar atoms of the benzimidazole carboxamide scaffold in its binding to CHK2 and we postulated that alternative binding modes may better explain this SAR. We therefore set out to explore alternative CHK2 binding modes for the benzimidazole class by application of unconstrained rigid docking, flexible side chain docking and protein–ligand crystallography.

In silico docking of small molecules into protein binding sites has become a powerful tool for medicinal chemistry design^27,28^; however, many docking protocols treat the protein as a rigid entity and, given the inconsistencies between reported SAR and the proposed benzimidazole binding modes in CHK2, we explored whether the introduction of side chain flexibility into the docking protocol would produce binding modes more consistent with the experimental SAR. A number of methods have been developed to introduce protein flexibility into docking protocols.^29–32^ One approach is to interrogate multiple crystal structures of the same protein;^33,34^ however, relatively few cases exist for which multiple crystal structures representing different protein conformations are available. Alternatively, computational methods can be applied to simulate protein conformational flexibility (e.g. by molecular dynamics);^35–38^ however, such methods are computationally intensive and artefact binding site conformations may be invoked. An alternative, and computationally less intensive approach, is to introduce protein side chain flexibility during the ligand docking process. A number of such methods have been developed^39^ and can be grouped into ‘knowledge-based’ and ‘induced-fit’ approaches.^40,41^ Knowledge-based approaches sample known side chain conformations from multiple crystal structures of a particular protein, which again requires their availability. Induced-fit approaches allow selected side chains in a single protein structure to move within a predefined range to sample a continuum of side chain conformations during ligand docking. We applied best practice unconstrained rigid docking and an induced-fit flexible docking protocol to establish the preferred binding mode of the benzimidazole inhibitor class in CHK2 and compared this predicted binding mode with the crystal structures of four exemplar benzimidazole inhibitors bound to CHK2.

## 2 Results and discussion

### 2.1 Analysis of literature CHK2 protein–ligand crystal structures

**Figure 2:**
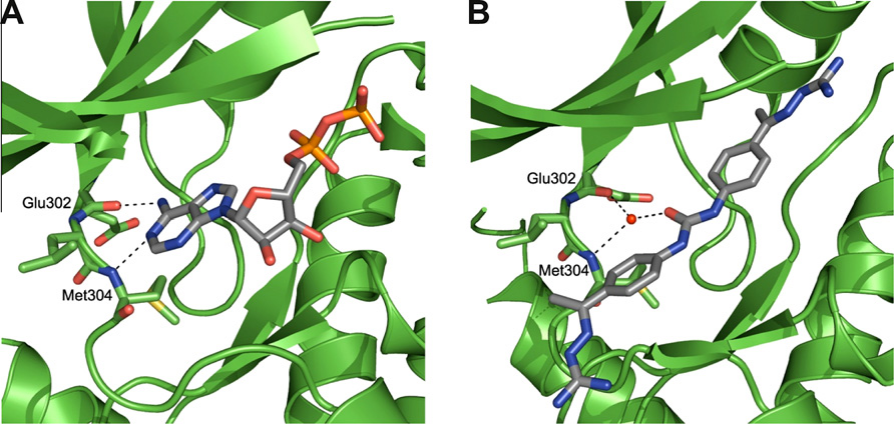
Binding of (A) ADP to CHK2 (PDB ID: 2CN5)^42^ and (B) NSC109555 to CHK2 (PDB ID: 2W0J).^43^ Hydrogen bonds between the ligand and the hinge region are indicated with black dashed lines.

We divided the 19 publicly available CHK2 protein–ligand crystal structures into two groups. The first comprises crystal structures in which the ligand mimics ADP and interacts with the hinge region of CHK2 through one or two hydrogen bonds between the ligand and residues Glu302 and Met304 (Fig. 2A).^42^ The second comprises 9 crystal structures with ligands such as NSC109555 (Fig. 2B)^43^ and closely related derivatives, for example PV1019,^10^ in which interaction with the hinge is mediated through a bound water molecule. The rmsd of this conserved water molecule between the 9 crystal structures is 0.62 Å. The binding mode of ADP corresponds to one of the closely related hinge binding modes postulated for the benzimidazole series (Fig. 1A).^16^ Intriguingly, the postulated benzimidazole binding mode, which involves a bidentate hydrogen bond donor and acceptor interaction only to Met304 (Fig. 1B), is not represented in the currently available CHK2 protein–ligand crystal structures.

### 2.2 Rigid docking of benzimidazole inhibitors

We carried out best practice unconstrained rigid docking using a selected set of 50 potent compounds from the published benzimidazole series (Table S1).^15–17^ We chose the structures of CHK2 bound to ADP (PDB ID: 2CN5) and to NSC1095555 (PDB ID: 2W0J) as representative high resolution parent structures for docking [2.25 and 2.05 Å, respectively]. To validate the suitability of these structures for docking, ligands were removed and re-docked into the empty structures using GOLD.^44^ In the NSC109555-bound structure the water molecule mediating interaction of the inhibitor with the hinge was retained. In both cases the ligand poses with the best scores had the same binding mode and interactions as observed in the respective crystal structures.

**Figure 3:**
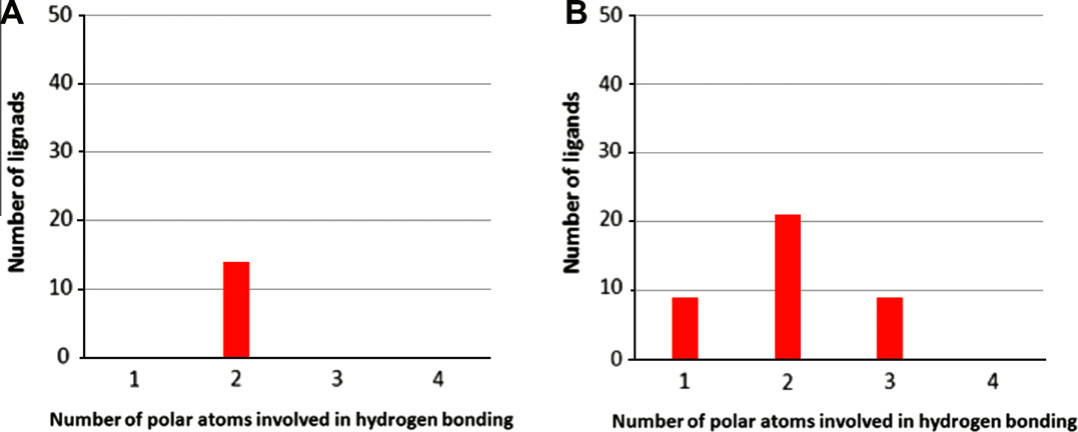
Number of polar interactions formed by: (A) Ligands which docked via the hinge to the apo-structure derived from the ADP-bound structure (PDB ID: 2CN5) (B). Ligands which docked via the mediating water molecule to the apo-structure derived from the NSC-109555-bound structure (PDB ID: 2W0J).

We subsequently docked the selected set of 50 active benzimidazole inhibitors (Table S1) and an inactive control set of inhibitors (compounds **8**–**12**, Table 1) into the two CHK2 structures using the interaction with the hinge residues (Glu302 and Met304) as a key requirement for a good solution. None of the inactive control set of inhibitors bound in either protein conformation. In the structure representing the ADP-bound CHK2 conformation (PDB ID: 2CN5), hydrogen bond interactions with both Glu302 and Met304 were observed for the lowest energy poses of 14 active compounds (compounds: **1, 13**, **15**, **16**, **18**, **19**, **20**, **22**, **30**, **33**, **38**, **39**, **47** and **59**); the other 36 compounds did not make these interactions. However, even for the 14 ligands that did bind to the hinge, no additional hydrogen bonds with the protein were identified from the Protein–Ligand Interaction Fingerprint (PLIF, Fig. 3). By contrast, 39 of the 50 compounds were found to bind via the mediating water molecule using the CHK2 conformation derived from the NSC109555-bound crystal structure. However, additional polar contacts between the inhibitor and the protein, as defined by the PLIF, were suboptimal for all 39 compounds (Fig. 3).

In summary, an unconstrained rigid docking protocol yielded a higher hit rate when applied to the NSC109555-derived protein structure 2W0J versus the ADP-derived protein structure 2CN5; however, neither protein structure provides binding mode solutions consistent with the importance of interactions from all 4 polar atoms of the benzimidazole–carboxamide scaffold, as demonstrated by the experimental SAR (*vide supra* and Table 1).

### 2.3 Flexible docking of benzimidazole inhibitors

We hypothesised that rigid docking may preclude the optimal orientation of protein side chains in the ATP binding site and applied a docking protocol incorporating side chain flexibility to both binding modes of CHK2 exemplified by protein structures 2W0J and 2CN5. Flexibility of up to 10 protein side chains can be explored using the commercial program GOLD.^44,45^ Limiting the number of residue side chains treated as flexible minimises the generation of false positive results whilst allowing a significant number of protein conformations to be generated. However, care has to be taken to ensure an objective choice of residues treated as flexible. In brief, we first applied a distance cut-off, based upon our rigid docking results for protein structures 2CN5 and 2W0J, to include all protein residues of the respective ligand-binding site with the potential to be treated as flexible. This criterion recognises that significant protein conformational change and associated energetic penalties are incurred for repositioning side chains distant from the ligand. Secondly, proximal glycine and alanine residues were deselected because they have no flexible side chain. Thirdly, we reasoned that, during the docking process, only residue side chains would be treated as flexible and interactions with the protein backbone would be unlikely to influence the outcome. Therefore, proximal residues interacting only through their backbone atoms were not selected for side chain flexibility. Fourthly, residues which have their side chain pointing away from the ligand were deselected, again recognising that significant protein conformational changes and associated energetic penalties are incurred for repositioning of side chains distant from the ligand. Application of these criteria reduced the number of selected residues to 16 and 27 in the 2CN5 and 2W0J structures, respectively (Table S2, Supplementary data). Defining objective criteria for residue selection in flexible docking protocols has been reported to be difficult,^31,32^ and consistent with this literature precedent, the remaining residues were manually inspected and residues with side chain flexibility impaired by hydrogen bonds and/or hydrophobic interactions with neighbouring residues were deselected. Finally, residues deeply buried in the binding pocket were prioritised over those on the protein surface until the limit of 10 was obtained. After application of these criteria, Cys231, Val234, Lys249, Glu308, Asp347, Glu351, Asn352, Asp368, Leu354 and His371 in the ADP-bound structure 2CN5; and Leu226, Val234, Lys249, Leu301, Glu308, Asp311, Leu354, Gln358, Thr367 and Asp368 in the NSC109555-bound structure 2W0J were assigned to be flexible during the docking runs.

The set of 50 biochemically active benzimidazole inhibitors was docked into the two parent CHK2 structures allowing side chains of the ten selected residues in each structure to flex. For the ADP-derived CHK2 conformation, 40 compounds were predicted to bind to the hinge region via hydrogen bonds between the benzimidazole–carboxamide and both Glu302 and Met304. Of these compounds, 18 were predicted to form one or more additional hydrogen bonds to the protein (Table S3, Supplementary data). For the NSC109555-derived CHK2 conformation, 27 compounds were predicted to interact with the hinge via the mediating water molecule; of these, 24 compounds were predicted to form one or more additional hydrogen bonds with the protein (Table S4, Supplementary data).

### 2.4 Rigid docking into ligand-induced protein conformations

**Figure 4:**
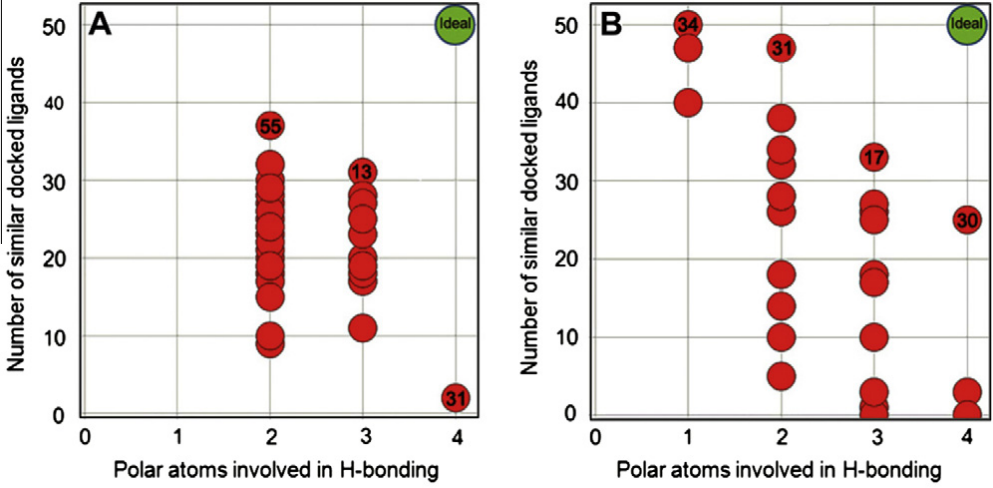
Trade-off surface for selecting the optimal ligand-induced ADP-derived protein conformation (A) and the optimal ligand-induced NSC109555-derived protein conformation (B). Each red dot denotes a different ligand-induced protein structure and numbers in bold within the dot denote the ligand used to generate the ligand-induced structure. For each plot, the *x*-axis denotes the number of polar atoms of the scaffold involved in hydrogen bonding in the respective ligand-induced structure; the *y*-axis denotes the number of docked ligands in the respective ligand-induced structure which show 0–4 polar interactions.

To objectively prioritise the multiple resultant ligand-induced protein conformations, we docked the dataset of 50 biochemically active ligands (Table S1, Supplementary data) into each ligand-induced protein conformation using an unconstrained rigid docking protocol. We reasoned firstly, that use of a ligand-induced protein conformation in a subsequent rigid docking protocol should deliver a similar binding mode for compounds similar to the docked ligand; and secondly that the binding mode of a ligand obtained using flexible docking should be reproduced by rigid docking into the protein conformation induced by that ligand. We then analysed the trade-off between the number of polar interactions formed in a ligand-induced binding mode and the number of docked ligands adopting that particular binding mode (Fig. 4 and Supplementary Tables S3 and S4). We recognise that polar interactions are only one component of the total protein–ligand interaction energy; however, optimisation of such interactions, and minimisation of unsatisfied ligand H-bond valencies which incur desolvation penalties, are also significant drivers of ligand efficient binding and, in this case, is consistent with the observed SAR. We selected optimal solutions closest to the trade-off surface and where multiple solutions lay close to the surface, preference was given to those with the highest number of polar atoms involved in hydrogen bonding.

**Figure 5:**
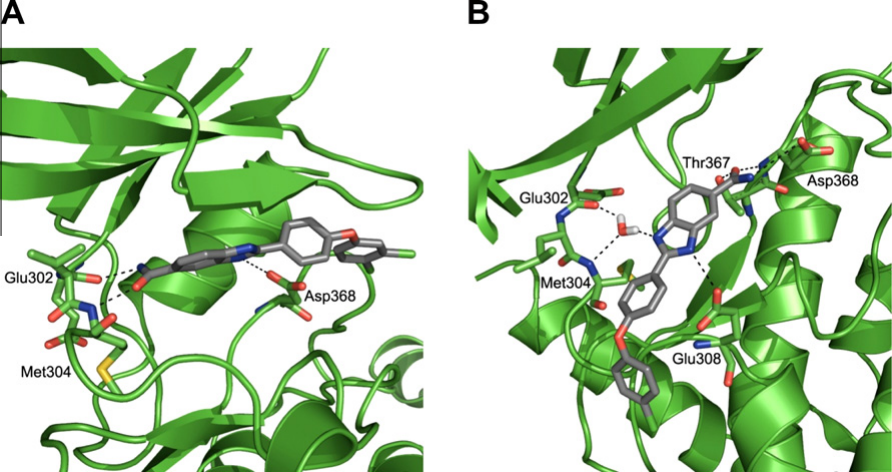
Ligand **13**-induced protein conformation (A) in which the benzimidazole carboxamide forms hydrogen bonds with Glu302 and Met304 and ligand **30**-induced protein conformation (B) in which a hydrogen bond is formed to the water molecule. Hydrogen bonds are depicted as dashed black lines.

For the ADP-derived CHK2 protein conformation, the protein conformation induced by ligand **13** has the optimal balance of a high number of docked ligands (30 out of a possible 50) that form a high number of hydrogen bonds (3 out of a possible 4) (Fig. 4A). The three polar interactions observed in the ligand **13-**induced conformation are the two hydrogen bonds to the hinge (Glu302 and Met304) and an interaction between the carboxylate side chain of Asp368 and the N3 atom of the benzimidazole scaffold (Fig. 5A). For the NSC109555-derived CHK2 protein conformation, the protein conformation induced by ligand **30** has the optimal balance of a high number of docked ligands (25 out of a possible 50) that form the highest possible number of hydrogen bonds (4 out of a possible 4) (Fig. 4B). The ligand **30**-induced conformation reveals hydrogen bonds between the conserved water molecule to nitrogen N1 as well as residue Glu308 to nitrogen N3 of the benzimidazole scaffold and interactions from the primary carboxamide moiety to side chains Thr367 and Asp368 which are adjacent to the catalytic lysine residue Lys249 (Fig. 5B). Docking of the negative control set (**8** to **12,** Table 1) into the ligand **13**- or ligand **30**-induced structures showed that none of these ligands achieved four hydrogen bonds to the scaffold in either induced structure.

Considering all possible solutions, these results indicate that the ligand **30**-induced conformation provides the optimal compromise of a high number of hydrogen bonds (4 out of a possible 4) adopted by a high number of biochemically active ligands (50%) docked into this ligand-induced protein conformation. Thus, the introduction of side chain flexibility into the docking protocol delivers an optimal binding mode mediated by a conserved water molecule to the hinge region. This binding mode is consistent with the observed experimental SAR (vide infra) and is significantly different from the two closely related binding modes previously postulated in the literature.^15–17^

**Figure 6:**
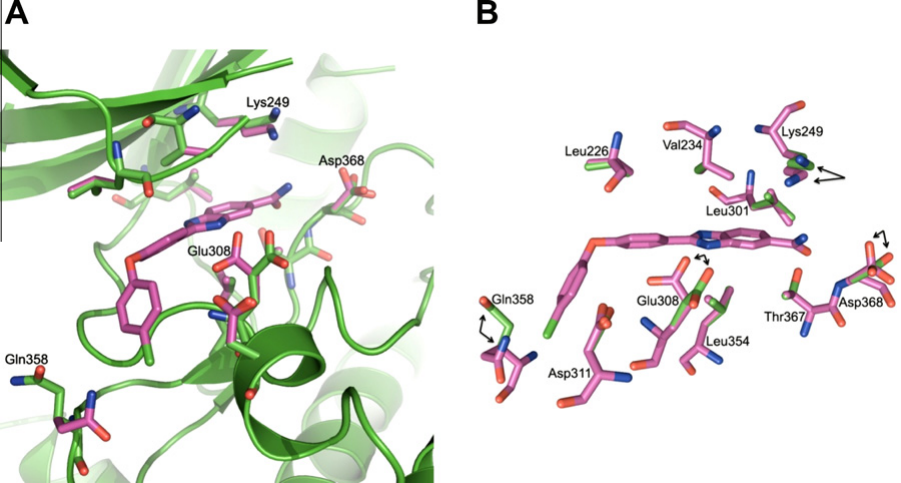
Overlay of the flexible residues in the binding sites of the NSC109555-bound crystal structure (green, PDB ID: 2W0J) and ligand **30**-induced protein structure (purple). Shown is (A), the CHK2 binding site highlighting residues which adopt a different conformation in the ligand **30**-induced protein structure (purple) and (B), a cut away depiction highlighting residues which adopt a different conformation in the ligand **30**-induced protein structure (purple) versus the parent crystal structure (green, PDB ID: 2W0J); arrows indicate side chains that have changed conformation significantly. The ligand pose is taken from the ligand **30**-induced conformation.

We compared the optimal ligand **30**-induced conformation with the crystal structure 2W0J (Fig. 6). Of the 10 residues treated as flexible, all hydrophobic protein side chains are aligned similarly in both the ligand **30**-induced conformation and in 2W0J. However, polar residues Lys249, Asp311 and Glu308 differ in their side chain conformation and, particularly in the case of Glu308, facilitate the formation of optimal hydrogen bonding interactions in the ligand-induced structure (Fig. 6). Taken together, these results demonstrate that flexible side chain docking reveals polar side chain interactions important to the ligand binding mode and consistent with the observed SAR. Should flexible polar side chains change conformation most significantly in ligand-induced models, then selection of residues for flexible docking could be restricted to proximal polar residues.

### 2.5 CHK2 protein–ligand crystallography

**Figure 7:**
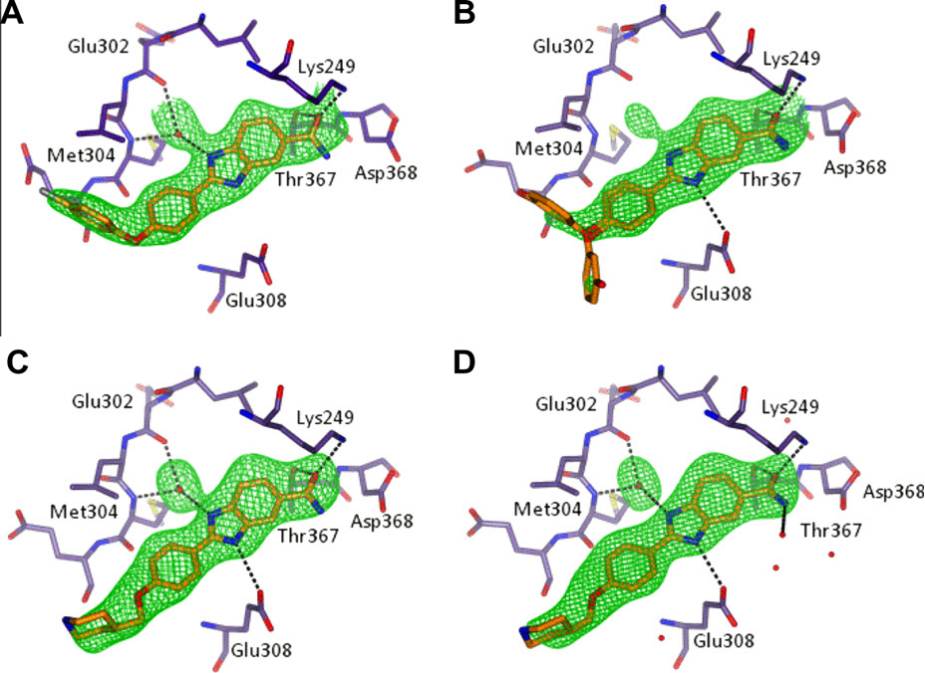
Crystal structures of CHK2 kinase in complex with inhibitors: (A) compound **30**, (B) compound **19**, (C) compound **44** and (D) compound **47**. The protein is shown in ribbon format, including cylinder representation for residues within 4 Å of the inhibitor (or the water molecule bridging the inhibitor and the hinge). An omit map showing Fo-Fc density is shown in green and contoured at 3.0σ. While strong electron density was present in each dataset for the 2-(4-oxyphenyl)-1*H*-benzo[*d*]imidazole-5-carboxamide scaffold, the weak density for the phenolic substituent of compound **19** (Panel B) suggested two alternate conformations for this moiety. The benzyl and chlorobenzyl groups of compound **44** and **47** (Panels C and D) were completely disordered, and have not been modelled. Electron density corresponding to the water molecule bridging the benzimidazole and the hinge region was observed in all four structures, but, after refinement, was omitted from the model for compound **19** (Panel B). Proposed H-bonds are indicated by black dotted lines. A summary of the data collection and refinement statistics is presented in Supplementary data (Table S5).

To validate the output from ligand-induced flexible docking, we solved the crystal structures of CHK2 co-crystallised with compounds **19**, **30**, **44** and **47**. In all four protein–ligand structures, electron density for the benzimidazole scaffold is well defined and unambiguously shows that the observed binding mode (Fig. 7) is similar to that reported for NSC109555 (Fig. 2). All four inhibitors bind with the benzimidazole core sandwiched between Leu354 at the bottom and Val234 (located in the P-loop) in the ceiling of the ATP pocket. The phenyl ring on the benzimidazole 2-position is positioned between the loop extending from the hinge region and Leu226 of the P-loop. The amide moiety interacts via its oxygen atom with the side chains of Thr367 and Lys249. The main differences between ligands are observed in their solvent exposed regions. In ligand **30,** the chlorophenyl group loosely binds in a hydrophobic pocket at the entrance of the active site defined by the side chains of Leu226, Leu236, Lys245, Leu303, Glu305 (Fig. 7A). The corresponding phenol in compound **19** is mainly disordered, but weak density suggests two main conformations (Fig. 7B). In the two most elaborated compounds, **44** and **47,** the respective benzyl and chlorobenzyl groups are completely disordered; consequently, these groups were not included in the final coordinate files (Fig. 7C and 7D). Minor differences in the interactions of the four ligands with CHK2 may be due to differences in data quality and include the presence of the hydrogen bond between the carboxamide NH_2_ and Asn352 in compound **19**, the absence of the hydrogen bond between the carboxamide NH_2_ and the side chain of Asp368 in compound **30** and **47**, and the absence of the hydrogen bond between the benzimidazole N3 atom and Glu308 in compound **19**. In crystal structures with compounds **30**, **44** and **47**, the water molecule interacting with the benzimidazole N1 atom and the hinge is present, as observed for NSC109555. Despite a small peak in the Fo-Fc electron density map corresponding to this water molecule for compound **19**, subsequent refinement led to a high B-factor and poor 2Fo-Fc density, so this water molecule was omitted from the final model. This may be attributable to the slightly lower resolution of this structure; however, electron density is well defined for both the ligand and protein hinge region.

**Figure 8:**
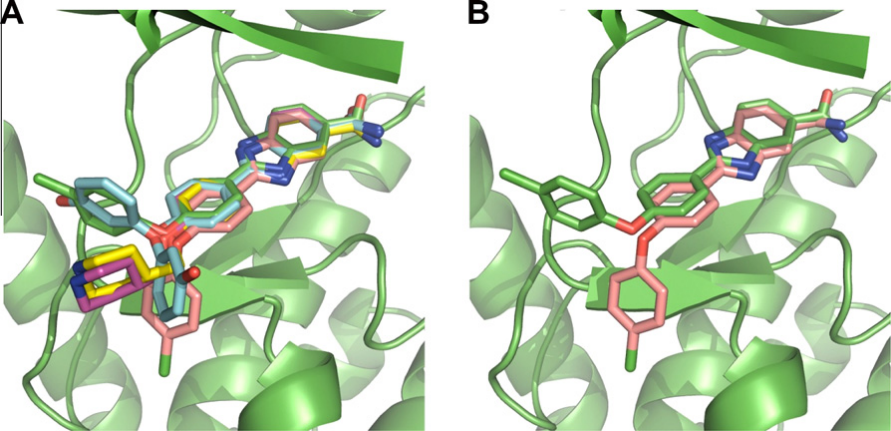
(A) Overlay of the ligand **30-**induced conformation (pink) and the CHK2 crystal structure of compound **19** (blue), compound **30** (green), compound **44** (purple) and compound **47** (yellow). (B) Overlay of the ligand **30-**induced conformation (pink) and the CHK2 crystal structure of compound **30** (green).

The binding mode for compound **30** predicted from flexible docking is in excellent agreement with the experimentally determined crystal structures for compounds **19**, **30**, **44** and **47** bound to CHK2 (Fig. 8A); the rmsd values for the conserved 2-(4-oxyphenyl)-1*H*-benzo[*d*]imidazole-5-carboxamide scaffold between the predicted binding mode for compound **30** and each of the four experimentally determined structures range from 0.41 to 0.67 Å. One difference is the orientation of the 4-chlorophenyl group of **30** (Fig. 8B); however, the two conformations of the 4-chlorophenyl observed in the X-ray of **19** suggests that both modes are possible (Fig. 8A). A second difference is a small rotation of the primary carboxamide with respect to the benzimidazole scaffold; however, a comparison of all four ligand-bound crystal structures reveals a range of amide conformations (Fig. 8A), suggesting that any rotational difference is within experimental error. We observed no significant differences between protein side chain conformations which define the active site across the four experimentally derived crystal structures, supporting the selection of a single protein conformation for compound docking. We used the CHK2 protein conformation, including the conserved water molecule, from the co-crystal structure of compound **30** to dock the set of 50 ligands (Supplementary Table S1) using a rigid docking protocol. 40 Compounds from the 50 ligand set formed all 4 hydrogen bonds from the benzimidazole scaffold in their docked poses consistent with the binding mode observed by X-ray crystallography.

### 2.6 SAR is consistent with the observed binding mode

**Figure 9:**
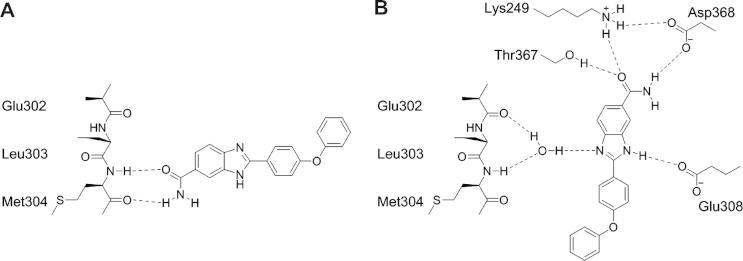
(A) Postulated binding mode of benzimidazole inhibitors^16^ and (B) schematic representation consistent with the binding mode observed by X-ray crystallography and including a water-mediated interaction of the benzimidazole scaffold with the hinge region.

The observed binding mode for the benzimidazole series in CHK2 is entirely consistent with the biochemical SAR data (Table 1). All polar atoms of the amide and benzimidazole form hydrogen bonds, consistent with the experimental observation that methylation of the benzimidazole N1 nitrogen atom (compound **8**, Table 1) ablates activity (Fig. 9). Methylation at the N3 position (compound **9**, Table 1) would be predicted to reduce but not abolish activity because, although the hydrogen bond interaction with Glu308 would be lost, the water mediated interaction with the hinge via N1 is maintained. A possible steric clash between the N3-methyl group and Glu308 is minimised by flexibility of the glutamic acid side chain. Replacement of the nitrogen at N3 by an oxygen atom (compound **11**, Table 1) would be predicted to lock the tautomeric forms of the scaffold and compromise activity through an unfavourable electrostatic clash with the carboxylic acid moiety of Glu308.

The water-mediated interaction between the benzimidazole N1 atom and the hinge region is clearly observed in three of the four protein ligand structures determined here, and there is evidence for electron density corresponding to this water molecule in the fourth structure; thus, this conserved water is an important determinant of the observed benzimidazole binding mode. Inclusion of this conserved water molecule in the NSC109555-derived protein conformation was, therefore, influential in the discovery of the novel benzimidazole binding mode by the in silico docking methods described here. Recent reports highlight the benefit of conserved water molecule inclusion in docking protocols for kinases and other gene families.^46,47^ The discovery of an optimal docking solution, consistent with the observed SAR, required inclusion of this conserved water molecule and application of a docking protocol incorporating the flexibility of polar protein side chains.

## 3 Conclusions

The binding mode for a series of benzimidazole inhibitors of the protein kinase CHK2 has been clarified by application of flexible side chain docking and protein–ligand crystallography. Although unconstrained rigid docking into the NSC109555-derived protein structure produces favourable docking solutions, none is consistent with the experimentally observed involvement of all four polar atoms of the carboxamido–benzimidazole scaffold in binding. However, the flexible side chain docking produces an optimal protein conformation and ligand binding mode that is entirely consistent with SAR from biochemical enzyme inhibition data. We observed that polar and flexible side chains: Lys249, Asp311, and particularly Glu308, change conformation most significantly in the ligand-induced model whilst hydrophobic residues are largely unchanged. The crystal structures of four exemplar benzimidazole inhibitors bound to CHK2 all show a single binding mode which is in excellent agreement with that obtained through flexible docking, but different from the prediction of unconstrained rigid docking, and significantly different from the binding modes previously postulated in the literature. This experimentally confirmed binding mode to the hinge region of CHK2 through a conserved water molecule has previously been observed for NSC109555,^43^ but has not previously been seen for a benzimidazole scaffold, and may provide a useful approach to selective small molecule inhibitor design for CHK2.

## 4 Materials and methods

### 4.1 Compound selection

To assess the performance of side chain flexible docking, active compounds were selected from a published set of CHK2 inhibitors with a benzimidazole scaffold, which were all characterised using the same biochemical assay protocol.^15–17^ Compounds were defined as active if their biochemical IC_50_ was 100 nM or less. Ligands with an ambiguous stereochemical assignment were removed from the dataset leaving 50 compounds for analysis (compounds **1** and **13** to **61**, Table S1, Supplementary data). To enable validation of the flexible docking results, a set of 5 compounds reported to be inactive, or show significantly reduced activity in the CHK2 biochemical screen, was used as a negative control group (compounds **8** to **12**, Table 1).

### 4.2 Ligand preparation

The benzimidazole core scaffold has two tautomeric forms both of which were generated for docking studies. A preferred lowest-energy 3D conformation was obtained for each compound using Corina.^48^ The protonation state of compounds was assigned using OpenEye Filter.^49^

### 4.3 Protein crystal structure preparation

CHK2 crystal structures (PDB ID: 2CN5 and 2W0J) representing different ligand binding modes were obtained from the Protein databank. To optimize the positioning of hydrogen atoms in the ATP binding pocket, each crystal structure was subject to Protonate3D as implemented in MOE^50^ using the default settings. Subsequently, the ligand and water molecules not interacting with the hinge were removed.

### 4.4 Rigid docking

Unconstrained rigid docking was performed using GOLD.^44^ For each compound, the number of binding poses generated was set to 20. The search space was defined by locating the centre of the ligand present in each crystal structure (ADP for structure 2CN5 and NSC109555 for structure 2W0J) and using a radius of 16 Å from the centroid to define the volume of the binding pocket searched during docking. To ensure that all possible binding modes were explored, early termination of the docking run as a consequence of multiple solutions with the same binding mode was disabled. Scoring of the binding poses was performed using GOLDscore.^45^ The 20 top scoring poses were subject to further analysis (see Section 4.6).

### 4.5 Flexible side chain docking

Unconstrained flexible side chain docking was performed using GOLD with the number of side chains treated as flexible limited to ten. For each protein crystal structure, an overlay was generated of each pose from Rigid Docking which passed our PLIF filter (see Section 4.6). The following residue selection criteria were then applied in sequence:

1. Side chains with all heavy atoms outside a radius of 3.5 Å from the nearest heavy atom of any ligand in its corresponding rigid docking pose were deselected.^31^
2. Glycine and alanine residues were deselected because they do not have a flexible side chain.
3. Residues interacting with the ligand only via their backbone atoms were deselected.
4. Residues with side chains pointing away from the ligand were deselected.

Consistent with literature precedent that defining objective criteria for residue selection is difficult^31,32^ further residues were deselected after manual inspection to prioritise 10 residues using the following criteria:

1. Residues with side chain flexibility impaired by hydrogen bonds and/or hydrophobic interactions with neighbouring residues were deselected.
2. Residues were prioritised from those deeply buried in the binding pocket (highest priority) to those on the protein surface (lowest priority) until the limit of 10 was achieved.

The permitted amount of flexibility for the 10 selected residues was defined by the parameters *Crystal* and *Library* in GOLD as described by Lovell et al.^51^ These settings allowed the side chains to flex around their initial positions with a maximal flexibility in line with the most commonly observed side chain conformations of naturally occurring amino acids.^48^ The search space was defined as for rigid docking. For each compound, 20 protein-inhibitor complexes were generated and scoring of the interaction between the inhibitor and the induced protein conformation was performed using GOLDscore.^45^ Early termination of the docking run, due to the generation of multiple solutions with the same binding mode, was disabled. The 20 top scoring poses were subject to further analysis (see Section 4.6).

### 4.6 Analysis of docking results

To identify the protein residues interacting with a docked inhibitor, the resulting protein-inhibitor complexes were analysed using the protein–ligand interaction fingerprint (PLIF) implemented in MOE, a method similar to the SIFt.^52,53^ The PLIF parameter ‘lower interaction threshold’ was set to 2% to allow the detection of weak hydrogen bonds between the ligand and the protein.^54^ The hinge-binding interaction was used as a rigid anchor and maintained in all compound-induced protein conformations. Compound poses that did not form this key interaction were discarded. The number of interactions for each compound pose with its induced protein model was manually scored using the PLIF (Supplementary Tables S3 and S4).

### 4.7 Selection of optimal ligand-induced protein conformations

Each ligand-induced protein conformation was analysed by plotting the trade-off between the number of polar interactions formed by the benzimidazole-5-carboxamide scaffold in the ligand-induced binding mode and the number of docked ligands adopting that particular binding mode (Fig. 5 and Tables S3 and S4). The selected ligand-induced protein conformation from this trade-off surface had the optimal combination of a high number of polar interactions and a high number of docked near neighbour ligands adopting that particular binding mode. This selected ligand-induced protein conformation was confirmed by application of unconstrained rigid docking using GOLD as described above.

### 4.8 Protein production and crystallography

The CHK2 kinase domain (CHK2-KD, amino acids 210-531) was produced as a GST-fusion protein and purified as previously described.^42^ Co-crystallisation experiments with four selected benzimidazole inhibitors were carried out based upon conditions described earlier.^12,13,42^ For full details of these experiments, crystallographic data collection and refinement see Supplementary data and Table S6.

### 4.9 Chemistry methods

Compound structures have previously been disclosed although preparative methods have not been described for all compounds.^15–17^ Materials and methods used to prepare compounds **19**, **30**, **44** and **47** for protein–ligand crystallography are described in Supplementary data.

### 4.10 Accession codes

Atomic coordinates and structure factors for the crystal structures of ligand-bound CHK2 can be accessed using the following PDB codes: **19**, 4A9S; **30**, 4A9R; **44**, 4A9T and **47**, 4A9U.

## Figures and Tables

**Table 1:** Structure activity relationships for the benzimidazole series of CHK2 inhibitors

No.	Structure	CHK2 IC_50_ (nM)
1		55 ± 31 ^15^
2		640 ± 210 ^15^
3		1900 ± 610 ^15^
4		1500 ± 720 ^15^
5		1400 ± 550 ^15^
6		>10000 ^15^
7		290 ± 90 ^15^
8		>10000 ^16^
9		1540 ^16^
10		>10000 ^16^
11		>10000 ^16^
12		>10000 ^16^

Numbering to denote the regiochemistry of benzimidazole methylation is shown for compounds **8** and **9**.

## Supplementary data

Supplementary data The ligands used in the flexible docking study table S1), the residue selection for side chain flexible docking (tables S2–S4) and experimental protocols for preparation of compounds studied by co-crystallography and associated crystallographic analysis.
