# Supplementary material for: Benzimidazole inhibitors of the protein kinase CHK2: Clarification of the binding mode by flexible side chain docking and protein–ligand crystallography
Source: Bioorg Med Chem. 2012 Nov 15;20(22):6630–9. doi: 10.1016/j.bmc.2012.09.024 (PMC3778940; doi:10.1016/j.bmc.2012.09.024)
Supplement: Supplementary data — The ligands used in the flexible docking study table S1), the residue selection for side chain flexible docking (tables S2–S4) and experimental protocols for preparation of compounds studied by co-crystallography and associated crystallographic analysis. [file mmc1.doc]

**Benzimidazole inhibitors of the protein kinase CHK2: Clarification of the binding mode by flexible side chain docking and protein-ligand crystallography.**

Cornelis Matijssen^1^, M. Cris Silva-Santisteban^1,2^, Isaac M. Westwood^1,2^, Samerene Siddique^1^, Vanessa Choi^1,2^, Peter Sheldrake^1^, Rob L.M. van Montfort^1,2^* and Julian Blagg^1^*

^1^Cancer Research UK Cancer Therapeutics Unit, Division of Cancer Therapeutics, Institute of Cancer Research, Haddow Laboratories, Sutton, Surrey SM2 5NG, UK

^2^Division of Structural Biology, The Institute of Cancer Research, Chester Beatty Laboratories, Chelsea, London SW3 6JB, UK

**Table S1.** Set of 50 ligands used in docking experiments.

|  | | | |
| --- | --- | --- | --- |
| **Ligand** | **R_1_** | **CHK2 IC_50_ (nM)** | **Reference** |
| **1** |  | 55 ± 31 | 1 |
| **13** |  | 5.5 ±1.1 | 1 |
| **14** |  | 4.4 ± 1.3 | 1 |
| **15** |  | 4.5 ± 2.1 | 1 |
| **16** |  | 12 ± 3.2 | 1 |
| **17** |  | 12 ± 3.8 | 1 |
| **18** |  | 47 ± 4.3 | 1 |
| **19** |  | 61 ± 31 | 1 |
| **20** |  | 9.8 ± 1.0 | 1 |
| **21** |  | 12 ± 1.1 | 1 |
| **22** |  | 17 ± 2.2 | 1 |
| **23** |  | 23 ± 9.5 | 1 |
| **24** |  | 24 ± 1.1 | 1 |
| **25** |  | 31 ± 8.9 | 1 |
| **26** |  | 81 ± 34 | 1 |
| **27** |  | 41 ± 34 | 1 |
| **28** |  | 18 ± 0.8 | 1 |
| **29** |  | 100 ± 26 | 1 |
| **30** |  | 16 ± 6.9 | 1 |
| **31** |  | 14 ± 4.1 | 1 |
| **32** |  | 3.4 ± 1.3 | 1 |
| **33** |  | 8.2 ± 2.0 | 1 |
| **34** |  | 13 ± 6.6 | 1 |
| **35** |  | 6.6 ± 3.0 | 1 |
| **36** |  | 32 ± 4.4 | 1 |
| **37** |  | 2.3 ± 0.5 | 1 |
| **38** |  | 52 ± 4 | 2 |
| **39** |  | 73 ± 7 | 2 |
| **40** |  | 37 ± 3 | 2 |
| **41** |  | 57 ± 10 | 2 |
| **42** |  | 55 ± 2 | 2 |
| **43** |  | 14 ± 8 | 2 |
| **44** |  | 70 ± 3 | 2 |
| **45** |  | 70 ± 16 | 2 |
| **46** |  | 67 ± 20 | 2 |
| **47** |  | 60 ± 8 | 2 |
| **48** |  | 45 ± 5 | 2 |
| **49** |  | 61 ± 25 | 2 |
| **50** |  | 74 ± 47 | 2 |
| **51** |  | 53 ± 42 | 2 |
| **52** |  | 90 ± 40 | 2 |
| **53** |  | 90 ± 5 | 2 |
| **54** |  | 95 ± 22 | 2 |
| **55** |  | 82 ± 22 | 2 |
| **56** |  | 39 ± 18 | 2 |
| **57** |  | 52 ± 51 | 2 |
| **58** |  | 23 ± 3 | 2 |
| **59** |  | 100 ± 20 | 2 |
| **60** |  | 78 ± 16 | 2 |
| **61** |  | 86 ± 10 | 2 |

**Table S2.** The number of residues deselected at each step of residue selection.

| Crystal structure used for selecting residues where side chains are allowed to flex during docking. | **2CN5** | **2W0J** |
| --- | --- | --- |
| Number of residues with at least one heavy atom within a 3.5 Å radius of any ligand heavy atom (from Rigid Docking) | 26 | 35 |
| Number of Ala or Gly residues deselected | 6 | 5 |
| Number of residues deselected which interact with the ligand only *via* their backbone atoms | 2 | 0 |
| Number of residues deselected which have their side chain pointing away from the ligand | 2 | 3 |
| Number of residues deselected because of the limited ability of the side chain to change conformation | 6 | 12 |
| Number of residues deselected because the side chain is solvent exposed | 0 | 5 |

**Table S3.** Results from rigid docking of the 50 compound set into the 40 ligand-induced protein conformations based upon the ADP-bound crystal structure 2CN5.

| Protein conformation induced by ligand **No.** | Number of hydrogen bonds formed with the benzimidazole carboxamide scaffold in the corresponding ligand-induced conformation | Number of polar atoms of the benzimidazole carboxamide scaffold involved in hydrogen bonding in the corresponding ligand-induced conformation. | Number of ligands (from the set of 50) which interact with two hydrogen bonds to the hinge (Glu302, Met304) upon rigid docking into the ligand-induced protein conformation. | Number of docked ligands (from the set of 50) which show the same number of benzimidazole carboxamide polar atoms involved in hydrogen bonding as the parent ligand. |
| --- | --- | --- | --- | --- |
| **1** | 3 | 3 | 21 | 20 |
| **13** | 3 | 3 | 30 | 30 |
| **14** | 3 | 3 | 17 | 17 |
| **15** | 2 | 2 | 31 | 31 |
| **16** | 3 | 3 | 28 | 28 |
| **17** | 2 | 2 | 29 | 29 |
| **19** | 3 | 3 | 28 | 26 |
| **20** | 3 | 3 | 28 | 24 |
| **21** | 3 | 3 | 24 | 24 |
| **22** | 2 | 2 | 8 | 8 |
| **23** | 2 | 2 | 31 | 31 |
| **24** | 3 | 3 | 14 | 11 |
| **25** | 2 | 2 | 18 | 18 |
| **26** | 3 | 3 | 26 | 22 |
| **27** | 2 | 2 | 25 | 25 |
| **28** | 2 | 2 | 19 | 19 |
| **29** | 2 | 2 | 17 | 17 |
| **30** | 3 | 3 | 20 | 19 |
| **31** | 4 | 4 | 15 | 2 |
| **32** | 3 | 3 | 18 | 17 |
| **33** | 3 | 3 | 21 | 17 |
| **34** | 3 | 3 | 25 | 23 |
| **35** | 3 | 3 | 21 | 18 |
| **36** | 2 | 2 | 26 | 26 |
| **37** | 2 | 2 | 14 | 14 |
| **38** | 3 | 3 | 28 | 27 |
| **39** | 2 | 2 | 21 | 21 |
| **40** | 2 | 2 | 25 | 25 |
| **41** | 2 | 2 | 21 | 21 |
| **42** | 2 | 2 | 19 | 19 |
| **44** | 2 | 2 | 24 | 24 |
| **45** | 2 | 2 | 9 | 9 |
| **47** | 2 | 2 | 26 | 26 |
| **48** | 3 | 3 | 12 | 11 |
| **49** | 2 | 2 | 27 | 27 |
| **51** | 2 | 2 | 26 | 26 |
| **55** | 2 | 2 | 36 | 36 |
| **57** | 3 | 3 | 27 | 25 |
| **59** | 2 | 2 | 24 | 24 |
| **60** | 2 | 2 | 28 | 28 |

**Table S4.** Results from rigid docking of the 50 compound set into the 27 ligand-induced protein conformations based upon the NSC109555-bound crystal structure 2W0J.

| Protein conformation induced by ligand **No.** | Number of hydrogen bonds formed with the benzimidazole carboxamide scaffold in the corresponding ligand-induced protein conformation | Number of polar atoms of the benzimidazole carboxamide scaffold involved in hydrogen bonding in the corresponding ligand-induced protein conformation. | Number of ligands (from the set of 50) which interact with the hinge via the mediating water molecule upon rigid docking into the ligand-induced protein conformation. | Number of docked ligands with the same number of benzimidazole carboxamide polar atoms involved in hydrogen bonding as the parent ligand |
| --- | --- | --- | --- | --- |
| **1** | 2 | 2 | 36 | 14 |
| **13** | 3 | 3 | 50 | 18 |
| **14** | 3 | 3 | 42 | 26 |
| **15** | 3 | 3 | 41 | 16 |
| **16** | 5 | 4 | 47 | 3 |
| **17** | 4 | 3 | 50 | 33 |
| **18** | 3 | 3 | 50 | 26 |
| **19** | 4 | 4 | 42 | 0 |
| **20** | 2 | 2 | 45 | 31 |
| **22** | 4 | 3 | 50 | 10 |
| **23** | 3 | 3 | 36 | 25 |
| **24** | 3 | 3 | 43 | 0 |
| **25** | 2 | 2 | 41 | 9 |
| **26** | 2 | 2 | 50 | 25 |
| **29** | 5 | 3 | 39 | 1 |
| **30** | 5 | 4 | 50 | 25 |
| **31** | 3 | 2 | 48 | 46 |
| **32** | 2 | 2 | 50 | 34 |
| **33** | 2 | 2 | 31 | 5 |
| **34** | 1 | 1 | 49 | 49 |
| **35** | 3 | 3 | 50 | 0 |
| **36** | 2 | 2 | 47 | 27 |
| **38** | 1 | 1 | 39 | 39 |
| **39** | 2 | 2 | 50 | 38 |
| **49** | 1 | 1 | 46 | 46 |
| **60** | 3 | 3 | 49 | 3 |
| **61** | 2 | 2 | 50 | 17 |

**Chemistry Experimental**

**General Methods**

Chemicals and solvents (including anhydrous solvents) were purchased from commercial sources and were used as received unless otherwise stated. Reactions were followed by thin layer chromatography (TLC) using pre-coated aluminium sheets of silica 60 F_254_ (Merck Art. 5735), or by LCMS using a Micromass LCT/Waters Alliance 2795 separations module HPLC system with a Merck Chromolith SpeedROD RP-18e 50 x 4.6 mm column at a temperature of 22 °C. The following solvent system, at a flow rate of 2 mL/min, was used: solvent A: methanol; solvent B: 0.1% formic acid in water.  Gradient elution was as follows: 1:9 (A:B) to 9:1 (A:B) over 2.25 min., 9:1 (A:B) for 0.75 min. then reversion back to 1:9 (A:B) over 0.3 min., 1:9 (A:B) for 0.2 min. Detection was carried out with a Waters 2487 Dual λ Absorbance Detector (detecting at 254nm) and ionisation was electrospray (ESI). Organic solutions were dried over sodium sulphate and were evaporated at reduced pressure. Flash column chromatography was carried out using BDH ‘Silica Gel for flash chromatography’ (purchased from VWR International Ltd.). NMR spectra were recorded on a Bruker Ultrashield 500MHz Avance instrument. HRMS analyses were performed on a Agilent 1200 series HPLC system with a Merck Chromolith SpeedROD RP-18e 50 x 4.6 mm column at a temperature of 22 °C. The following solvent system, at a flow rate of 2 mL/min, was used: solvent A: methanol; solvent B: 0.1% formic acid in water.  Gradient elution was as follows: 1:9 (A:B) to 9:1 (A:B) over 2.5 min., 9:1 (A:B) for 1 min. then reversion back to 1:9 (A:B) over 0.3 min., 1:9 (A:B) for 0.2 min. This was connected to a Agilent 6200 Time of Flight (ToF) mass spectrometer (simultaneous ESI and APCI or ESI only) with detection at 254nm. The following reference masses were used for HRMS analysis: caffeine [M + H]+ = 195.087652, reserpine [M + H]+ = 609.280657 and (1*H*,1*H*,3*H*-tetrafluoropentoxy)phosphazene [M + H]+ = 922.009798. The purity of final compounds was determined by HPLC as described above and is ≥ 95% unless specified otherwise.

**Preparative Procedures**

Samples of compounds **19, 30, 44** and **47** were prepared as shown in Schemes S1, S2, S3 and S4 respectively. Compounds **19** and **30** have been described and characterised in the literature.^1^ Compounds **44** and **47** have been described but not characterised in the literature.^2^

**Compound 19**

**Scheme S1**: Synthetic route to compound **19**

4-(3-Methoxyphenoxy)benzaldehyde **62**

A mixture of 3-methoxyphenol (325 mg, 2.62 mmol), 4-fluorobenzaldehyde (330 mg, 2.66 mmol) and potassium carbonate (424 mg, 3.07 mmol) in *N*,*N*-dimethylacetamide (3 mL) was stirred and heated at 160^o^C in the microwave for 1 hour. A second batch was run at the same scale and the two combined for workup. The reactions were cooled, added to water (55 mL) and the mix extracted with ethyl acetate (3 x 20 mL). The combined extracts were washed with water (3 x 15 mL) and with brine, dried (Na_2_SO_4_) and evaporated. The crude product was purified on a flash column (silica, eluting with 5% ethyl acetate in 60-80 petroleum and with 10% ethyl acetate in 60-80 petroleum) to give pure product (520 mg, 41%). ^1^H-NMR (CHCl_3_, 500MHz): 3.81 (s, MeO, 3H); 6.65 (m, 1H); 6.68 (m, 1H); 6.78 (m, 1H); 7.09 (d J = 8.83 Hz, 2H); 7.31 (t, J = 8.05 Hz, 1H); 7.86 (d, J = 8.83 Hz, 2H); 9.94 (s, CHO, 1H).

2-(4-(3-Methoxyphenoxy)phenyl)-1*H*-benzo[*d*]imidazole-5-carboxamide **63**

To a mixture of 4-amino-3-nitrobenzamide (162 mg, 0.89 mmol) and 4-(3-methoxyphenoxy) benzaldehyde (204 mg, 0.89 mmol) in ethanol (6.2 mL) was added a freshly made 1M solution of sodium dithionite (3.1 mL, 3.1 mmol) and the reaction was stirred and heated at 75^o^C for 4 hours. The reaction was cooled and most of the ethanol was evaporated. Water (5 mL) was added. The solid was filtered, washed twice with water and dried in a vacuum desiccator over sodium hydroxide to leave a solid (306 mg, 95%). ^1^H-NMR (d_6_-DMSO, 500MHz): 3.76 (s, MeO, 3H); 6.67 (d, J = 7.88 Hz, 1H); 6.71 (s, 1H); 6.80 (d, J = 8.20 Hz, 1H); 7.19 (d, J = 8.51 Hz, 2H); 7.32 (s, NH_2_, 1H); 7.34 (t, J = 8.20 Hz, 1H); 7.61 (d, J = 8.51 Hz, 1H); 7.80 (d, J = 8.51 Hz, 1H); 8.01 (s, NH_2_, 1H,); 8.15 (s, 1H); 8.20 (d, J = 8.83 Hz, 2H).

2-(4-(3-Hydroxyphenoxy)phenyl)-1*H*-benzo[*d*]imidazole-5-carboxamide **19**^1^

2-(4-(3-Methoxyphenoxy)phenyl)-1*H*-benzo[*d*]imidazole-5-carboxamide (53 mg, 0.15 mmol) was stirred in dichloromethane (3 mL). To the suspension was added a 1M solution of boron tribromide in dichloromethane (0.6 mL, 0.6 mmol). The reaction was stirred at room temperature for 2.5 hours. Water (3 mL) was added and stirred for 1 hour. The liquids were decanted leaving a gum, which was washed with water (2 x 4 mL). Ethanol (5 mL) was added and the resulting solution was evaporated. The residue was triturated with ether to give a grey solid (47 mg). This was applied in 1:1 chloroform : methanol to four 1 mm 20 x 20 cm silica preparative TLC plates, which were eluted with 20:1 ethyl acetate : methanol. The product band was recovered with 1:1 ethyl acetate : methanol. The residue was azeotroped with ethanol to give the product (26 mg, 52%). ^1^H-NMR (d_6_-DMSO, 500 MHz): 6.47 (t, J = 2.21 Hz, 1H); 6.51 (dd, J = 8.20 Hz, 2.21 Hz, 1H); 6.61 (dd, J = 8.20 Hz, 2.21 Hz, 1H); 7.15 (d, J = 8.83 Hz, 2H); 7.20 (t, J = 8.20 Hz, 1H); 7.25 (s, NH, 1H,); 7.58 (d, J = 8.20 Hz, 1H); 7.75 (d, J = 8.20 Hz, 1H); 7.97 (s, NH, 1H); 8.13 (s, 1H); 8.22 (d, J = 8.83 Hz, 2H); 13.40 (s, br, NH, 1H).

ESI-HRMS: Found: 346.11885, calculated for C_20_H_16_N_3_O_3_ (M+H)^+^: 346.11862.

**Compound 30**

**Scheme S2**: Synthetic route to compound **30**

4-(4-Chlorophenoxy)benzaldehyde **64**

A mixture of 4-chlorophenol (286 mg, 2.22 mmol), 4-fluorobenzaldehyde (276 mg, 2.22 mmol) and potassium carbonate (400 mg, 2.90 mmol) in *N,N*-dimethylacetamide (2.4 mL) was stirred and heated at 160^o^C in the microwave for 1 hour. The reaction was cooled, added to water (25 mL) and the mix extracted with ethyl acetate (3 x 12 mL). The combined extracts were washed with water (3 x 10 mL) and with brine, dried (Na_2_SO_4_) and evaporated to leave the product (507 mg, 98%). ^1^H-NMR (CHCl_3_, 500MHz): 7.04 (d, J = 8.51 Hz, 2H); 7.07 (d, J = 8.83 Hz, 2H); 7.38 (d, J = 8.51 Hz, 2H); 7.87 (d, J = 8.51 Hz, 2H); 9.94 (s, CHO, 1H).

4-Amino-3-nitro-benzamide **65**^1^

To a solution of 4-amino-3-nitrobenzoic acid (2.50 g, 13.7 mmol) and 1-hydroxybenzotriazole (2.05 g, 15.1 mmol) in THF (125 mL) was added 1-(3-dimethyaminopropyl)-3-ethylcarbodiimide hydrochloride (2.90 g, 15.1 mmol). Diisopropylethylamine (2.65 mL, 1.95 g, 15.1 mmol) was added and stirred at ambient temperature for 10 minutes. Ammonium carbonate (3.95 g, 41 mmol) was added and stirred at ambient temperature for 20 hours. The THF was evaporated and half-saturated sodium hydrogen carbonate solution (100 mL) was added and stirred for 1.5 hours. The yellow solid was filtered off and washed twice with a small volume of water. The product was dried in a vacuum desiccator over sodium hydroxide (2.39 g, 96%).

2-(4-(4-Chlorophenoxy)phenyl)-1*H*-benzo[*d*]imidazole-5-carboxamide **30**

To a mixture of 4-amino-3-nitrobenzamide (85 mg, 0.47 mmol) and 4-(4-chlorophenoxy)benzaldehyde (109 mg, 0.47 mmol) in ethanol (3.3 mL) was added a freshly made 1M solution of sodium dithionite (1.65 mL, 1.65 mmol) and the reaction was stirred and heated at 78^o^C for 6 hours, then stirred at ambient temperature overnight. Most of the ethanol was evaporated and water (3 mL) was added. The solid was filtered, washed twice with water and dried in a vacuum desiccator over sodium hydroxide to leave a solid (154 mg). An aliquot of the solid (52 mg) was applied in 1:1 chloroform : methanol to four 1 mm 20 x 20 cm silica preparative TLC plates. These were eluted with 10:1 ethyl acetate : methanol and the product band recovered with 1:1 ethyl acetate : methanol. The solution was evaporated and the residue triturated with ether to give a solid (33 mg, 57%). ^1^H-NMR (d_6_-DMSO, 500MHz): 7.15 (d, J = 8.83 Hz, 2H); 7.19 (d, J = 8.51 Hz, 2H); 7.26 (s, NH, 1H); 7.49 (d, J = 8.83Hz, 2H); 7.58 (d, J = 8.20 Hz, 1H); 7.76 (d, J = 8.20 Hz, 1H); 7.98 (s, NH, 1H); 8.14 (s, 1H); 8.23 (d, J = 8.51 Hz, 2H); 13.23 (s, br, NH, 1H).

ESI-HRMS: Found: 364.0843, calculated for C_20_H_15_ClN_3_O_2_ (M+H)^+^: 364.0847.

**Compound 44**

**Scheme S3**. Synthetic route to compound **44**

4-(1-*t*-Butoxycarbonylpiperidin-4-ylmethoxy)benzaldehyde **66**

To a solution of 4-hydroxybenzaldehyde (403 mg, 3.31 mmol), N-Boc-piperidine-4-methanol (711 mg, 3.31 mmol) and triphenylphosphine (867 mg, 3.31 mmol) in THF (7 mL) cooled to 0-5^o^C was added a solution of diisopropyl azodicarboxylate (669 mg, 3.31 mmol) in THF (3 mL) over 1 hour (syringe drive). The reaction was allowed to come to room temperature and was stirred for 25 hours. The THF was evaporated and the residue taken up in ethyl acetate (25 mL). The solution was washed with 0.2 M sodium hydroxide (10 mL), with 0.1 M sodium hydroxide (10 mL), with water and with brine; dried (Na_2_SO_4_) and evaporated to leave an oil (2.83 g). This was purified by flash chromatography (silica; eluting with 5% ethyl acetate in dichloromethane, 10% ethyl acetate in dichloromethane and 15% ethyl acetate in dichloromethane). Product-containing fractions were evaporated to give the product (608mg 57%). ^1^H-NMR (CHCl_3_, 500MHz):1.30 (m, 2H); 1.48 (s, 9H); 1.84 (d, J = 13.24 Hz, 2H); 2.00 (m, 1H); 2.76 (br, 2H); 3.9. 0 (d, J = 6.63Hz, 2H); 4.19 (br, 2H); 7.00 (d, J = 8.51 Hz, 2H); 7.84 (d, J = 9.14 Hz, 2H); 9.90 (s, CHO, 1H).

4-Piperidin-4-ylmethoxybenzaldehyde **67**

4-(1-*t*-Butoxycarbonylpiperidin-4-ylmethoxy)benzaldehyde (346 mg, 1.08 mmol) was dissolved in dichloromethane (3 mL) and trifluoroacetic acid (3 mL) was added and stirred at room temperature for 2 hours. The solvents were evaporated and saturated sodium bicarbonate solution was added. The product was extracted with ethyl acetate (10 then 8 then 7 mL) and the combined extracts were washed with brine, dried (Na_2_SO_4_) and evaporated to leave the amine (187 mg, 78%). ^1^H-NMR (CHCl_3_, 500MHz): 1.69 (q of d, J = 2.84 Hz, 12.30 Hz, 2H); 2.04 (d, J = 14.5 Hz, 2H); 2.11 (m, 1H); 2.90 (t of d, J = 2.84 Hz, 12.93 Hz, 2H); 3.43 (d, J = 12.61 Hz, 2H); 3.94 (d, J = 6.31 Hz, 2H); 7.00 (d, J = 8.83 Hz, 2H); 7.86 (d, J = 8.83 Hz, 2H); 9.90 (s, CHO, 1H).

4-(1-Benzylpiperidin-4-ylmethoxy)benzaldehyde **68**

4-Piperidin-4-ylmethoxybenzaldehyde (187 mg, 0.85 mmol) was dissolved in chloroform (4 mL) and benzaldehyde (92 μL, 95 mg, 0.90 mmol) was added followed by acetic acid (54 μL, 0.90 mmol) and sodium triacetoxyborohydride (192 mg, 0.90 mmol). The reaction was stirred at room temperature for 20 hours. The solvent was evaporated and the residue partitioned between 10% sodium carbonate solution (5 mL) and ethyl acetate (10 mL). The layers were separated and the aqueous again extracted with ethyl acetate (10 mL). The combined extracts were washed with brine, dried (Na_2_SO_4_) and evaporated to leave an oil (221 mg). This was applied in chloroform to two 2 mm, 20 x 20 cm silica preparative TLC plates, which were eluted with 2.5% 2 M ammonia in methanol, in ether. The product band was recovered with 2.5% 2 M ammonia in methanol, in ethyl acetate. The residue was azeotroped with ethanol to give the product (97 mg, 37%). ^1^H-NMR (CHCl_3_, 500MHz): 1.50 (m, br, 2H); 1.85 (m, br 3H); 2.09 (br, 2H); 3.01 (br, 2H); 3.59 (s, CH_2_Ph, 2H); 3.90 (d, J = 5.99 Hz, CH_2, 2H_); 6.99 (d, J = 8.51 Hz, 2H); 7.29 (m, 1H); 7.36 (m, 4H); 7.84 (d, J = 8.83 Hz, 2H); 9.89 (s, CHO, 1H).

2-(4-((1-Benzylpiperidin-4-yl)methoxy)phenyl)-1*H*-benzo[*d*]imidazole-5-carboxamide **44**

4-((1-Benzylpiperidin-4-yl)methoxy)benzaldehyde (95 mg, 0.31 mmol) and 4-amino-3-nitrobenzamide (50 mg, 0.27 mmol) were stirred in ethanol (1.9 mL) and a freshly made 1M solution of sodium dithionite (0.95 mL, 0.95 mmol) was added. The reaction was stirred and heated at 75^o^C for 18 hours. The reaction was cooled and 5 M ammonia solution (0.5 mL, 2.5 mmol) was added. The suspension was replaced by a gum beneath a clear supernatant. Water (1.5 mL) was added and on stirring the gum was converted to a solid. This was stirred for 30 minutes, filtered and washed with a little 1:1 ethanol : water. The solid was dried in a vacuum desiccator over sodium hydroxide leaving the crude product (84 mg, 74%). The solid was slurried with ethyl acetate (2 mL) for 80 minutes, then filtered and washed with ethyl acetate (2 mL). Drying gave the product (65 mg, 55%).

ESI-HRMS: Found: 441.2277, calculated for C_27_H_29_N_4_O_2_ (M+H)^+^: 441.2285

^1^H-NMR (d_6_-DMSO, 500MHz): two benzimidazole NH signals (12.94, 12.96 of equal intensity) suggests two tautomers (**44**-T1 and **44**-T2) which exist in d_6_-DMSO solution in a 1:1 ratio.

Using 1D and 2D NMR experiments, the NMR signals can be assigned to the two tautomers although many signals overlap.

|  | Proton Chemical Shift | | Carbon Chemical Shift | |
| --- | --- | --- | --- | --- |
| Carbon No | Tautomer 1 | Tautomer 2 | Tautomer 1 | Tautomer 2 |
| 1 |  | - | 127.9 | 127.9 |
| 2 | 7.78 | 7.74 | 122.2 | 121.3 |
| 3 | 7.51 | 7.63 | 110.4 | 117.6 |
| 4 |  | - | 137.2 | 146.2 |
| 5 |  | - | 143.7 | 134.6 |
| 6 | 8.21 | 8.03 | 118.1 | 110.9 |
| 8 |  | - | 152.9 | 153.3 |
| 10 | - | | 122.2 | |
| 11/15 | 8.13 | | 128.3 | |
| 12/14 | 7.11 | | 114.8 | |
| 13 | - | | 160.4 | |
| 17 | 3.91 | | 72.2 | |
| 18 | 1.77 | | 35.3 | |
| 23/19a | 1.34 | | 28.5 | |
| 23/19b | 1.76 | | 28.5 | |
| 22/20a | 1.97 | | 52.8 | |
| 22/20b | 2.85 | | 52.8 | |
| 24 | 3.47 | | 62.3 | |
| 25 | - | | 138.6 | |
| 26/30 | 7.30 | | 128.8 | |
| 27/29 | 7.33 | | 128.1 | |
| 28 | 7.24 | | 126.8 | |
| 31 | - | | 168.3 | |

**Compound 47**

**Scheme S4**: Synthetic route to compound **47**

1-(4-Chlorobenzyl)piperidine-4-methanol **69**

Piperidine-4-methanol (444 mg, 3.86 mmol) was dissolved in chloroform (8 mL) and 4-chlorobenzaldehyde (542 mg, 3.86 mmol) was added, followed by acetic acid (230 μL, 230 mg, 3.9 mmol) and sodium triacetoxyborohydride (900 mg, 4.24 mmol). The reaction was stirred at room temperature for 4 days. Solvent was evaporated and the residue partitioned between 0.4 M hydrochloric acid (20 mL) and ethyl acetate (20 mL). The layers were separated and the acid layer again washed with ethyl acetate (10 mL). The combined acid layers were basified with 2M sodium hydroxide (to about pH 10) and the product was extracted with ethyl acetate (3 x 20 mL). The combined extracts were washed with brine, dried (Na_2_SO_4_) and evaporated to leave the product (722 mg, 83%). ^1^H-NMR (CDCl_3_, 500 MHz): 1.29 (q of d, J = 3.89 Hz, 12.30 Hz, 2H); 1.50 (m, 1H), 1.72 (m, 2H); 1.80 (br, OH, 1H); 1.96 (t of d, J = 2.69 Hz, 11.82 Hz, 2H); 2.88 (m, 2H); 3.47 (s, 2H); 3.50 (d, J = 6.62 Hz, 2H); 7.27 (m, 4H).

4-((1-(4-Chlorobenzyl)piperidin-4-yl)methoxy)benzaldehyde **70**

To a solution of 1-(4-chlorobenzyl)piperidine-4-methanol (374 mg, 1.56 mmol), 4-hydroxybenzaldehyde (191 mg, 1.56 mmol) and triphenylphosphine (409 mg, 1.56 mmol) in THF (4 mL) cooled in ice-water was added a solution of diisopropyl azodicarboxylate (315 mg, 1.56 mmol) in THF (1 mL) over 10 minutes. The reaction was stirred at 0-5^o^C for 30 minutes and at room temperature for 18 hours. Solvent was evaporated and ethyl acetate (15 mL) added. The solution was washed with 0.2M sodium hydroxide (2 x 8 mL), with water and with brine. Drying (Na_2_SO_4_) and evaporation left a heavy oil (1.18 g). This was purified on a flash column (silica, eluting with ethyl acetate) to give the desired product mixed with triphenylphosphine oxide (482 mg). This mixture was applied in chloroform to three 2 mm 20 x 20 cm silica preparative TLC plates, which were eluted with ether. The product band was recovered with acetone to give the product (81 mg, 15%). ^1^H-NMR (CHCl_3_, 500MHz): 1.25-1.29 (m, 1H); 1.39-1.48 (m, 2H); 1.80-1.85 (m, 2H); 1.99-2.05 (m, 2H); 2.90-2.95 (m, 2H); 3.49 (s, 2H); 3.89 (d, J = 5.99 Hz, 2H); 6.99 (d, J = 8.51 Hz, 2H); 7.25-7.31 (m, 4H); 7.84 (d, J = 8.51 Hz, 2H); 9.89 (s, CHO, 1H).

2-(4-((1-(4-Chlorobenzyl)piperidin-4-yl)methoxy)phenyl)-1*H*-benzo[d]imidazole-5-carboxamide **47**

4-((1-(4-Chlorobenzyl)piperidin-4-yl)methoxy)benzaldehyde (76 mg, 0.22 mmol) and 4-amino-3-nitrobenzamide (40 mg, 0.22 mmol) were stirred in ethanol (1.6 mL) and a freshly made 1M solution of sodium dithionite (0.8 mL, 0.8 mmol) was added. The reaction was stirred and heated at 80^o^C for 6 hours. Most of the ethanol was evaporated and water (3 mL) was added. The solid was filtered off, washed with water and dried overnight in a vacuum desiccator over sodium hydroxide to leave a solid (105 mg). An aliquot of this solid (52 mg) was applied in 1:1 chloroform : methanol (heat and ultrasound needed to get a solution) to four 1 mm 20 x 20 cm silica preparative TLC plates. These were eluted with 200:30 ethyl acetate : 2 M ammonia in methanol and the product band recovered with 1:1 ethyl acetate : 2 M ammonia in methanol. The residue was triturated with ether to give the title compound (29 mg, 56%). ESI-HRMS: Found: 475.1885, calculated for C_27_H_28_ClN_4_O_2_ (M+H)^+^: 475.1895. ^1^H-NMR (d_6_-DMSO, 500 MHz): two benzimidazole NH signals (13.00, 13.01 equal intensity) suggests two tautomers. The exchange rate of the two tautomers (**47**-T1 and **47**-T2) causes broadening of the NMR spectra when **47** is dissolved in d_6_-DMSO. Using 2D NMR experiments, most carbons can be assigned. Multiple conformations of the piperidine ring caused ^13^C signal broadening.

| Carbon No | Proton Chemical Shift | Carbon Chemical Shift |
| --- | --- | --- |
| 1 | - | 129.65 |
| 2 | 7.84 | 123.54 |
| 3 | 7.67 | 115.14 |
| 4 | - | NA |
| 5 | - | NA |
| 6 | 8.21 | 116.23 |
| 8 | - | 155.50 |
| 10 | - | 123.31 |
| 13 | - | 162.40 |
| 17 | 4.02 | 72.50 |
| 18 | 2.15 | 34.60 |
| 24 | 4.32 | 60.70 |
| 25 | - | NA |
| 28 | - | 136.94 |
| 31 |  | 172.30 |
| 11/15 | 8.12 | 129.70 |
| 12/14 | 7.14 | 116.20 |
| 22/20a | 3.02 | 52.80 |
| 22/20b | 3.51 | 52.80 |
| 23/19a | 1.78 | 27.00 |
| 23/19b | 2.09 | 27.00 |
| 26/30 | 7.60 | 134.20 |
| 27/29 | 7.57 | 129.90 |

**Crystallography**

Co-crystallisation experiments were carried out at 4 ºC using the hanging-drop vapour diffusion method. Hanging drops were generated by mixing 2 μL protein solution, which typically consisted of 10 mg/mL CHK2-KD, 10 mM HEPES pH 7.5, 250 mM NaCl, 10 mM DTT, 2 mM EDTA and 2 mM of the inhibitor with 2% (*v/v*) DMSO, with 2 μL of precipitant solution composed of 0.1 M HEPES pH 7.6, 0.2 M Mg(NO_3_)_2_, 10% (*v/v*) ethylene glycol, 1 mM TCEP and either 8%, 12% or 14% (*w/v*) PEG 3350. The hanging drops were subsequently placed over 1 mL of precipitant solution. Crystals were harvested and cryo-protected using a cryoprotectant solution containing 0.1 M HEPES NaOH pH 7.5, 0.1 M NaCl, 0.2 M Mg(NO_3_)_2_, 20% (*v/v*) ethylene glycol and 10% (*w/v*) PEG 3350 before flash-freezing in liquid nitrogen.

All data sets were collected at beamline I03 at the Diamond Light Source (Oxfordshire, UK) and integrated, merged and scaled using the programs MOSFLM^5^ and SCALA from the CCP4 program suite.^6,7^ The data collection and refinement statistics are presented in Table S5. The four protein-ligand structures were solved by molecular replacement using Phaser^8^ and a CHK2-inhibitor complex (PDB code 2WTJ) with the inhibitor and water molecules removed as a search model. Difference maps were used to identify, and model, the position of the bound inhibitor. Protein-ligand structures were manually rebuilt in Coot^9^ and refined with phenix.refine^10^ in iterative cycles. Only weak electron density for the phenol group in compound **19** was observed, and the optimum electron density maps were obtained by modeling two alternate conformations for the phenol moiety of this ligand. The respective phenyl and chlorophenyl moieties in compound **44** and compound **47** were completely invisible in the electron density maps, therefore we only modeled the visible parts of these compounds. Translation, libration and screw (TLS) parameters and Molprobity structure validation tools were used within the Phenix environment.^11,12^

| **Table S5. Data collection and refinement statistics** | | |  |  |
| --- | --- | --- | --- | --- |
| **PDB Code** | **4A9S** | **4A9R** | **4A9T** | **4A9U** |
| **Compound** | **Compound 19** | **Compound 30** | **Compound 44** | **Compound 47** |
| Spacegroup | *P*3_2_21 | *P*3_2_21 | *P*3_2_21 | *P*3_2_21 |
| **Lattice constants** |  |  |  |  |
| *a,b* (Å) | 90.71 | 90.84 | 91.01 | 91.07 |
| *c* (Å) | 93.28 | 93.35 | 93.50 | 93.41 |
|  |  |  |  |  |
| **Data collection** |  |  |  |  |
| Resolution range (Å) | 78.56-2.66 | 60.19-2.85 | 60.26-2.70 | 60.26-2.48 |
| (Highest resolution shell) | (2.80-2.66) | (3.00-2.85) | (2.85-2.70) | (2.61-2.48) |
| Number of reflections | 43783 (5128) | 43472 (6495) | 70274 (10249) | 68316 (10022) |
| Unique reflections | 12863 (1751) | 10659 (1553) | 12692 (1827) | 16275 (2330) |
| Completeness (%) | 98.4 (93.9) | 99.2 (100.0) | 99.9 (100.0) | 99.8 (100.0) |
| Multiplicity | 3.4 (2.9) | 4.1 (4.2) | 5.5 (5.6) | 4.2 (4.3) |
| *R*merge (%) | 5.9 (45.4) | 6.6 (43.9) | 8.4 (45.1) | 8.8 (43.9) |
| I/σ(I) | 8.3 (1.7) | 8.6 (1.7) | 6.7 (1.6) | 4.8 (1.7) |
| Mean(I/σ(I)) | 11.5 (2.2) | 13.0 (3.3) | 13.4 (3.7) | 10.4 (3.3) |
| Mosaicity (°) | 0.77 | 0.66 | 1.13 | 0.11 |
|  |  |  |  |  |
| **Refinement** |  |  |  |  |
| Resolution range (Å) | 40.10-2.66 | 40.14-2.85 | 40.92-2.70 | 40.93-2.48 |
| No. of amino acids | 282 | 285 | 289 | 287 |
| No. of water molecules | 28 | 18 | 51 | 88 |
| Other ligand molecules | Compound 19 (1) | Compound 30 (1) | Compound 44 (1) | Compound 47 (1) |
|  | NO_3_^2-^ (1) | NO_3_^2-^ (1) | NO_3_^2-^ (1) | NO_3_^2-^ (1) |
|  |  | Ethylene Glycol (1) | Ethylene Glycol (3) | Ethylene Glycol (9) |
|  |  |  |  | Cl^-^ (1) |
| *R*factor (%) | 18.6 | 18.4 | 18.9 | 18.4 |
| *R*free^a^ (%) | 23.4 | 23.5 | 21.8 | 21.4 |
| **R.m.s. deviations** |  |  |  |  |
| Bond lengths (Å) | 0.004 | 0.004 | 0.009 | 0.003 |
| Bond angles (°) | 0.741 | 0.667 | 0.804 | 0.613 |
| **Mean B factors (**Å^2^**)** |  |  |  |  |
| Protein | 71.9 | 67.3 | 55.1 | 55.0 |
| Inhibitor | 72.3 | 79.5 | 67.5 | 54.4 |
| Solvent | 58.2 | 52.5 | 55.0 | 51.1 |
|  |  |  |  |  |
| **Ramachandran plot** |  |  |  |  |
| Favoured (%) | 97.4 | 96.1 | 95.8 | 97.8 |
| Generously allowed (%) | 2.2 | 3.2 | 3.8 | 2.2 |
| Forbidden (%) | 0.4 | 0.7 | 0.4 | 0.0 |
|  |  |  |  |  |
|  |  |  |  |  |
| ^a^ The Free *R* factor, *R*free, was computed using 5% of the data assigned randomly and is the same for all four structures.  All data were collected with a Quantum ADSC detector on Beamline I03 at the Diamond Light Source, Oxfordshire, on March 6 2009. The wavelength used for data collection was 0.9763 Å and the oscillation angle was 1° for each dataset collected. For each crystal, the Matthews’ coefficient was between 3.40 and 3.43 Å^3^/Da, with a solvent content of 64%. | | | | |

**Supplementary References**

1. Arienti, K.L.; Brunmark, A.; Axe, F.U.; McClure, K.; Lee, A.; Blevitt, J.; Neff, D.K.; Huang, L.; Crawford, S.; Pandit, C.R.; Karlsson, LBreitenbucher, J.G. Checkpoint kinase inhibitors: SAR and Radioprotective Properties of a series of 2-Arylbenzimidazoles. *J. Med. Chem*. **2005**, *48*, 1873-1885.
2. Neff, D.K.; Lee-Dutra, A.; Blevitt, J.M.; Axe, F.U.; Hack, M.D.; Buma, J.C.; Rynberg, R.; Brunmark, A.; Karlsson, L.; Breitenbucher, J.G. 2-Aryl benzimidazoles featuring alkyl-linked pendant alcohols and amines as inhibitors of checkpoint kinase Chk2. *Bioorg. Med. Chem. Lett.* **2007**, *17*, 6467-6471.
3. McClure, K.J.; Huang, L.; Arienti, K.L.; Axe, F.U.; Brunmark, A.; Blevitt, J. Breitehbucher, J.G. Novel non-benzimidazole Chk2 kinase inhibitors. *Bioorg. Med. Chem. Lett.* **2006**, *16*, 1924-1928.
4. Yang, D.; Fokes, D.; Li, J.; Yu, L.; Baldino, C.M. A Versatile Method for the Synthesis of Benzimidazoles from o-Nitroanilines and Aldehydes in One Step via a Reductive Cyclisation. *Synthesis* **2005**, 47-56.
5. Leslie, A.G.W. Recent changes to the MOSFLM package for processing film and image plate data. *Joint CCP4 + ESF-EAMCB Newsletter on Protein Crystallography, No. 26.* **1992**.
6. Collaborative Computational Project, Number 4. The CCP4 Suite: Programs for Protein Crystallography. *Acta Cryst. D* **1994**, *50*, 760-763.
7. Winn, M.D.; Ballard, C.C.; Cowtan, K.D.; Dodson, E.J.; Emsley, P.; Evans, P.R.; Keegan, R.M.; Krissinel, E.B.; Leslie, A.G.W.; McCoy, A.J.; McNicholas, S.J.; Murshudov, G.N.; Pannu, N.S.; Potterton, E.A.; Powell, H.R.; Read, R.J.; Vagin, A.; Wilson, K.S. Overview of the CCP4 suite and current developments. *Acta Cryst. D* **2011**, *67*, 235-242.
8. McCoy, A.J.; Grosse-Kunstleve, R.W.; Adams, P.D.; Winn, M.D.; Storoni L.C.; Read, R.J. *Phaser* crystallographic software. *J. Appl. Cryst.* **2007**, *40*, 658-674.
9. Emsley, P.; Lohkamp, B.; Scott, W.G.; Cowtan, K. Features and Development of Coot. *Acta Cryst. D* **2010**, *66*, 486-501.
10. Afonine, P.V.; Grosse-Kunstleve, R.W.; Adams, P.D. The Phenix refinement framework. *CCP4 Newsletter July, Contribution 8.* **2005**.
11. Adams, P.D.; Afonine, P.V.; Bunkoczi, G.; Chen, V.B.; Davis, I.W.; Echols, N.; Headd, J.J.; Hung, L.W.; Kapral, G.J.; Grosse-Kunstleve, R.W.; McCoy, A.J.; Moriarty, N.W.; Oeffner, R.; Read, R.J.; Richardson, D.C.; Richardson, J.S.; Terwilliger, T.C.; Zwart, P.H. PHENIX: a comprehensive Python-based system for macromolecular structure solution. *Acta Cryst. D* **2010**, *66*, 213-221.
12. Chen, V.B.; Arendall, W.B.; Headd, J.J.; Keedy, D.A.; Immormino, R.M.; Kapral, G.J.; Murray, L.W.; Richardson, J.S.; Richardson, D.C. MolProbity: all-atom structure validation for macromolecular crystallography. *Acta Cryst. D* **2010**, *66*, 16-21.
